# Corrigendum: Critical exponents and scaling invariance in the absence of a critical point

**DOI:** 10.1038/ncomms14372

**Published:** 2017-01-17

**Authors:** N. Saratz, D. A. Zanin, U. Ramsperger, S. A. Cannas, D. Pescia, A. Vindigni

Nature Communications 7: Article number: 13611; DOI: 10.1038/ncomms13611 (2016); Published 12 05 2016; Updated 12 17 2017

The original version of this Article contained a typographical error in the spelling of the author S.A. Cannas, which was incorrectly given as S. Cannas. This has now been corrected in both the PDF and HTML versions of the Article.

